# Cannabinoids, Endocannabinoids, and Synthetic Cannabimimetic Molecules in Neuromuscular Disorders

**DOI:** 10.3390/ijms25010238

**Published:** 2023-12-23

**Authors:** Fabio Arturo Iannotti

**Affiliations:** Institute of Biomolecular Chemistry (ICB), National Research Council of Italy (CNR), 80078 Pozzuoli, NA, Italy; fabio.iannotti@icb.cnr.it

**Keywords:** neuromuscular disorders, cannabinoids, endocannabinoids, cannabimimetic molecules

## Abstract

Neuromuscular disorders (NMDs) encompass a large heterogeneous group of hereditary and acquired diseases primarily affecting motor neurons, peripheral nerves, and the skeletal muscle system. The symptoms of NMDs may vary depending on the specific condition, but some of the most common ones include muscle weakness, pain, paresthesias, and hyporeflexia, as well as difficulties with swallowing and breathing. NMDs are currently untreatable. Therapeutic options include symptomatic and experimental medications aimed at delaying and alleviating symptoms, in some cases supplemented by surgical and physical interventions. To address this unmet medical need, ongoing research is being conducted on new treatments, including studies on medical cannabis, endocannabinoids, and related molecules with cannabimimetic properties. In this context, a significant amount of knowledge about the safety and effectiveness of cannabinoids in NMDs has been obtained from studies involving patients with multiple sclerosis experiencing pain and spasticity. In recent decades, numerous other preclinical and clinical studies have been conducted to determine the potential benefits of cannabinoids in NMDs. This review article aims to summarize and provide an unbiased point of view on the current knowledge about the use of cannabinoids, endocannabinoids, and synthetic analogs in NMDs, drawing from an array of compelling studies.

## 1. Neuromuscular Diseases

**Figure 1:**
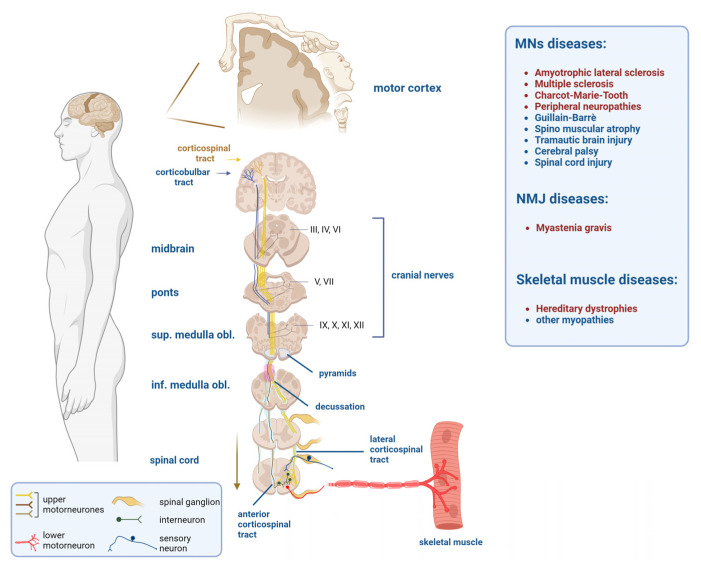
Illustration of the pyramidal (corticospinal and corticobulbar) tract. NMDs in which the use of cannabinoids is under investigation are in red.

Neuromuscular diseases (NMDs) are a diverse group of adult and pediatric disorders that affect approximately 15 million people worldwide. These diseases are caused by inherited or spontaneous mutations in over 500 autosomal or X-linked genes that encode components of the motor pathway, also known as the pyramidal tract. This pathway anatomically consists of the corticospinal and corticobulbar tracts [1]. The corticospinal tract is one of the most important descending tracts of the central nervous system (CNS). It is the pathway through which upper motor neurons (UMNs) originating from the motor and somatosensory cortex send their axons via the cerebral peduncle into the brainstem and anterior medulla oblongata. At this level, the majority of axons cross from one side to the other within structures called pyramids. Technically, this anatomical twist of somatic descending fibers is called decussation. The axons that decussate (approximately 80%) form the lateral corticospinal tract, with the remaining ones forming the anterior corticospinal tract (Figure 1). Once the UMN axons reach the spinal cord, they form synapses with lower motor neurons (LMNs) to innervate skeletal muscles, although the majority of synapses occur first with an interneuron in the anterior grey column [2] (Figure 1). The corticobulbar tract is instead formed by UMNs originating from the ventral motor cortex and terminates in the midbrain, pons, or medulla oblongata without passing through the pyramids (Figure 1). The somatic fibers within the corticobulbar tract are essential for transmitting motor signals to the cranial nerves that supply muscles of the head, neck, and face [3] (Figure 1).

Due to this complexity, NMDs are commonly sub-classified as disorders affecting motor neurons (MNs), spinal nerve roots, nerve plexuses, peripheral nerves, neuromuscular junctions (NMJ), and skeletal muscles [4] (Figure 1). The majority of NMDs are rare and often present symptoms that are similar to those of more common diseases. As a result, patients often experience a delayed diagnosis, and in the worst-case scenario (approximately 30% of cases), recovery it is never achieved, despite the significant progress made over the last few decades [5,6]. Clinical management and treatment of patients with NMDs are not less problematic. Major concerns arise when patients do not respond to initial treatments, or worse, when long-term therapies become ineffective, negatively impacting the quality and lifespan of patients [5,7]. This article discusses the successes, limitations, and hopes for future therapeutic intervention in NMDs, with a focus on the use of cannabinoids, endocannabinoids, or synthetic cannabimimetic analogs in the treatment of the disease. These conditions include amyotrophic lateral sclerosis, spinal muscular atrophy, Charcot–Marie–Tooth disease, peripheral neuropathies, myasthenia gravis, and Duchenne muscular dystrophy (Figure 1).

## 2. Cannabinoids, Endocannabinoids, and Synthetic Cannabimimetic Molecules

The term cannabinoids or phytocannabinoids commonly refers to ~120 or more compounds present in the trichomes of *Cannabis sativa*. Among them, Δ9-tetrahydrocannabinol (Δ^9^-THC or THC), cannabidiol (CBD), cannabichromene (CBC), and cannabigerol (CBG) are the most abundant, even though they are obtained only after a decarboxylation reaction achieved through heat from their acid precursors Δ^9^-THCA, CBDA, CBCA, and CBGA [8]. Within the body, cannabinoids produce their effects by simultaneously modulating multiple targets. For example, Δ^9^-THC, the psychoactive component of Cannabis, is known to act as a partial agonist of the cannabinoid receptors CB1 and CB2, while cannabidiol (CBD), the second most abundant phytocannabinoid, does not show a high affinity for CB1 or CB2 receptors, despite having the same molecular formula as Δ^9^-THC [9,10]. Thus, many other non-cannabinoid receptors are implicated, and in some cases, they are the primary actors in the mechanism of action of phytocannabinoids. Among them, we found members of large G-protein-coupled receptors (GPCR) including GPR55, GPR18, GPR3, GPR6, and GPR12. In addition, some types of ligand-gated ion channels, transient receptor potential channels (TRP), and intracellular classes of receptors (i.e., Peroxisome proliferator-activated receptors—PPARs) were demonstrated to be implicated in the biological effects of cannabinoids [11,12,13]. In this regard, the complex polyvalent pharmacological properties of phytocannabinoids are yet to be fully depicted and remain attractive in the context of searching for novel therapeutics against untreatable human diseases. In addition to the well-known beneficial effects of cannabinoids in alleviating chemotherapy-related nausea and vomiting, their use has expanded to treat other medical conditions such as chronic pain, cancer, anxiety, insomnia, epilepsy, and many others [14,15,16,17]. In the central nervous system (CNS), the ECS plays a major role in regulating neuronal excitability and synaptic plasticity through a mechanism known as “retrograde signaling”. This refers to the process by which during repetitive or tetanic stimulation, endocannabinoids are produced and released from the postsynaptic neuron to travel back to presynaptic terminals. At this site, endocannabinoids stimulate CB1 receptors, which in turn activate intracellular signaling to inhibit the release of major neurotransmitters such as glutamate, GABA, acetylcholine, norepinephrine, dopamine, and serotonin [18,19]. This mechanism, therefore, is responsible for the modulatory role played by the ECS in key neurological functions including eating, anxiety, stress, learning and memory, addiction-like behavior, and central pain sensation [20,21]. Retrograde signaling by endocannabinoids is also known to mediate short- and long-term synaptic plasticity in the spinal cord circuitry. In this context, Kettunen et al. in 2005 proved that the retrograde mechanism of endocannabinoids is activated in isolated lamprey spinal cord segments in response to mGluR1 stimulation. This activation contributes to the modulation of the locomotor burst frequency [22]. Additionally, a large body of evidence demonstrates that endocannabinoids, CB1 receptors, and other ECS-related proteins are expressed in key structures that regulate movement and motor coordination, including deep-layer cortical neurons, basal ganglia, and the cerebellum (see Fernández-Ruiz 2009 for review [23]).

Finally, the complex world of cannabinoids includes numerous synthetic compounds produced to mimic the activity of Δ^9^-THC- and/or endocannabinoids by directly regulating the two cannabinoid receptors (CB1 and CB2). This large class of molecules includes (a) synthetic potent agonists of CB1 such as AM-1235, arachidonic-2′ chloroethylamide (ACEA), JWH-073, and methanandamide; (b) synthetic potent agonists of CB2 such as JWH133, JWH015, HU-308, and AM1241; (c) mixed CB1/CB2 agonists such as WIN55,212-2, HU-210, and CP55,940; (d) selective antagonists such as Rimonabant/SR141716 and AM251 for CB1, and SR144528 and AM630 for CB2 [24]. Moreover, cannabimimetic agents include activators or inhibitors of the FAAH and MAGL, which are the two main enzymes responsible for the degradation of AEA and 2-AG, as well as agents that through indirect mechanisms change endocannabinoids synthesis and turnover (see Petrosino and Di Marzo for review [25].

## 3. Amyotrophic Lateral Sclerosis

Amyotrophic lateral sclerosis (ALS) is a fatal disease that often affects individuals aged 40 to 70, with a prevalence of approximately five cases per 100,000 people [26]. It leads to the gradual deterioration of both UMNs and LMNs in the brain and spinal cord to different extents. Signs of UMN damage include hyperreflexia, extensor plantar response, and increased muscle tone, while LMN signs include weakness, muscle wasting (atrophy), hyporeflexia, muscle cramps, and fasciculation [27]. In addition to motor neuron vulnerability, the disease’s clinical heterogeneity is expanded by the occurrence of extra-motor features, primarily consisting of cognitive and behavioral abnormalities that may emerge as the disease progresses or even precede the initial signs. The prevalent forms of ALS are sporadic (90%), while familial forms (10%) are mostly caused by mutations in genes encoding superoxide dismutase-1 (SOD-1), TAR-DNA binding protein-43 (TDP-43), fused in sarcoma (FUS), and C9orf72, even though mutations in numerous other genes have been associated with the disease. One of the most widely accepted hypotheses is that ALS results from a combination of genetic and epigenetic defects that are influenced by environmental factors [28]. Treatment options for ALS are limited. Riluzole was the first FDA-approved treatment for the disease in 1995. However, after two decades of research, it became clear that the drug provides only limited benefits to patients [29]. In recent years, two other medications, namely Edaravone (Radicava), a free radical scavenger and anti-oxidant, and AMX0035/Albrioza (Relyvrio), a combination of sodium phenylbutyrate and ursodoxicoltaurine (or taurursodiol), have been approved by the FDA, but not in Europe. Witzel et al. documented the poor long-term efficacy of edaravone compared to that of riluzole in a multicenter study published in JAMA Neurology in 2022 [30]. Similarly, Relyvrio, which appears to provide clinically significant benefits and extend the survival of ALS patients [31], has been rejected by the EU regulator once again this year.

The first study that examined the therapeutic potential of cannabinoids in ALS was conducted by Raman et al. in 2004. In brief, they found that treatment with Δ^9^-THC in SOD1 (G93A) transgenic mice prevented motor deficits and increased survival, whether administered before or after the onset of the disease [31]. One year later, Weydt et al. demonstrated that the phytocannabinoid CBN (cannabinol) also had significant benefits in delaying the onset of the disease in SOD1 (G93A) mice, although it did not extend their survival [32]. From these findings, numerous other studies have been conducted to explore whether or not cannabinoids, synthetic analogs, and modulators of the endocannabinoid system could potentially become new tools for complementary and alternative therapies in ALS, possibly through the identification of novel targets. Bilsland et al. found a significant delay in disease progression in 90-day-old SOD1 mice treated with WIN55212-2, a non-specific cannabinoid receptors agonist. Moreover, when the same mice were crossbred with those lacking the Faah gene (Faah^−/−^), which encodes for the main enzyme of AEA degradation, researchers observed a delay in the onset and severity of the disease. However, neither WIN55212-2 nor Faah ablation had a beneficial effect on the survival of mice. In addition, in SOD mice lacking the Cb1 gene (SOD Cnr1^−/−^), the same investigators found that the disease onset was not delayed, and conversely, survival was prolonged [33].

In the same year (2006), Kim and colleagues reported that the intraperitoneal injection of AM1241, a selective CB2 agonist, in SOD (G93A) mice was effective in slowing the signs of disease progression when administered after the onset of the disease [34]. Soon after, Shoemaker et al. demonstrated that the mRNA expression and receptor function of CB2, but not CB1, receptors were significantly upregulated in the spinal cords of SOD1 (G93A) mice in parallel with disease progression. They also found that AM-1241 was able to increase the mice’s survival [35]. Rodríguez-Cueto et al. discovered that VCE-003.2, a quinone derivative of the non-psychotropic phytocannabinoid cannabigerol (CBG), effectively mitigated most of the neuropathological symptoms in SOD1 mice. It also prevented the dysregulated expression of pro-inflammatory factors and glutamate transporters in cultured astrocytes obtained from the same mice [36]. Using the same animal model, Moreno-Martet et al. found a significant increase in CB2 receptors (only in female mice) and NAPE-PLD enzyme (responsible for AEA synthesis) in the spinal cords. Additionally, they reported that treatment with an equimolecular combination of Δ^9^-THC and CBD (20:20 mg/kg) resulted in a slight improvement in neurological signs and the survival rate in female mice [37]. Espejo-Porras et al. observed elevated levels of CB2 mRNA and protein in reactive microglial cells located in the spinal ventral horn of both early and post-symptomatic male and female TDP-43 transgenic mice, an alternative murine model of ALS. In contrast, the expression of CB1, as well as that of the other genes belonging to the endocannabinoid system (with the only exception of Faah), was unaltered. In addition, no changes were observed in AEA and 2-AG levels in the spinal cord of these animals [38]. Remarkably, in TDP-43 CB2 knock-out (TDP-43/Cnr2^−/−^) double-mutant mice, a faster decline in locomotor activities was observed, which was associated with an earlier death of motor neurons compared to that in control littermates [39]. Pharmacological experiments in TDP-43 mice revealed that HU-308, a synthetic selective CB2 agonist, significantly enhanced locomotor performance and preserved the survival of motor neurons in the ventral horn. However, WIN55212-2 had only modest beneficial effects, while treatment with AM630, a selective CB2 antagonist, did not significantly alter the course of the disease [38].

In summary, these studies highlighted the significant role of CB2 receptors, rather than CB1, in contributing to the progression and severity of ALS in mouse models of the disease. Most benefits can be achieved by activating CB2 receptors primarily in astrocytes and reactive microglial cells located in the dorsal and ventral horns of the spinal cord. Limited data from clinical studies are available on the benefits of cannabinoids or synthetic compounds in ALS. Weber and colleagues conducted a randomized, double-blind, placebo-controlled crossover trial with 27 patients (7 women and 20 men; mean age: 57 years) suffering from moderate to severe daily cramps. The trial found that orally administered THC (5 mg) twice daily was ineffective in reducing cramp intensity and preventing fasciculation intensity. No effect of THC was found on the quality of life, sleep, appetite, or mood [32]. In 2019, Riva et al. conducted a phase 2 study (CANALS) that presented initial evidence of the effectiveness and safety of Nabiximols (THC and CBD in a 1:1 ratio) compared to a placebo in reducing spasticity in ALS patients [40]. Meyer et al. reported data collected from a retrospective monocentric cohort study based on a platform-based patient registry, which supported the use of THC:CBD as a valuable option in managing ALS patients with moderate to severe spasticity [41]. In a recent study by Lacroix et al., 28 patients with ALS self-medicating with cannabidiol (CBD) oil and cannabis declared to have benefits in terms of both motor (rigidity, cramps, and fasciculations) and non-motor (sleep quality, pain, emotional state, quality of life, and depression) symptoms [42]. Overall, despite the promising findings from animal models, there is insufficient strong evidence to support the use of cannabinoids and cannabimimetic agents in humans. Last but not least, the existing literature suggests that the use of cannabis, whether with high or low Δ^9^-THC, may impair both voluntary and spontaneous movements by interfering with the cortico-striatal pathways. However, there is conflicting data on this issue (see Prashad for a review [43]).

## 4. Multiple Sclerosis

Multiple sclerosis (MS) is a chronic, disabling disease that affects the brain and spinal cord of young adults aged 20 to 40 years. The disease is caused by defects in the immune system, which overreacts against the myelin sheath wrapped around nerve fibers, thus disabling communication between the brain and the rest of the body [44]. The term “multiple sclerosis” refers to the development of multiple sclerotic areas caused by the immune system’s attack on myelin. Depending on the clinical course, the disease is classified into three main types: (a) Relapsing-Remitting MS (RRMS), (b) Primary-Progressive MS (PPMS), and (c) Secondary-Progressive MS (SPMS). The RR phenotype is the form manifested by the majority of patients (approximately 85%) who experience new and recurrent neurologic symptoms called “relapses”. These symptoms often fully disappear, only to reappear in an exacerbated manner, with no apparent progression of the disease during the periods of recovery. In contrast, the PPMS form affecting about 10–15% of MS patients is characterized by the progressive degeneration of neurological functions from the onset of symptoms, with no periods of remission. The third form of Secondary-Progressive MS often follows RRMS and is characterized by the gradual worsening of disability over time, independent of occasional relapses [45,46,47]. More uncommon forms of MS include (a) Progressive-Relapsing MS (PRMS); (b) Marburg variant; and (c) Balo’s concentric sclerosis. The factors influencing the various forms of MS as well as the causes are unknown. Consequently, a cure for MS is currently unavailable. Treatment options, however, may help reduce relapse rates and alleviate some of the most common symptoms, such as spasticity (affecting 60–85% of patients), pain (neuropathic and non-neuropathic), tremors, ataxia, bladder dysfunction, and sleep disorders [48].

The use of medical cannabis and cannabinoids in MS has generated significant interest, and numerous studies have been published on this topic in the last twenty years. The initial evidence of cannabinoids suppressing symptoms of MS was obtained by surveying 112 patients from two different countries (USA and UK) who self-medicated with cannabis. In brief, more than 90% of these patients reported benefits in managing spasticity, muscle pain, tremors, and depression [49]. Thereafter, seven clinical trials were conducted between the 1980s and 1990s with Δ^9^-THC. In five studies, Δ^9^-THC was administered orally in a dosage ranging from 5 to 15 mg. In addition, Maurer et al. evaluated the effect of Δ^9^-THC 5 mg (oral route) in MS patients taking baclofen and clonazepam, while Breneisen et al. performed an open-label study to assess the effect of Δ^9^-THC (oral route, 10–15 mg) compared to Δ^9^-THC hemisuccinate (rectal route, 10–15 mg) (see Pertwee 2002 for review [49]). Other clinical trials have been conducted using dronabinol (trade name: Marinol; synthetic Δ^9^-THC), nabilone (trade name: Cesamet; synthetic Δ^9^-THC analog), and nabiximols (trade name: Sativex; 1:1 THC:CBD). In summary, the clinical data are controversial, making it difficult to reach a definitive conclusion about the therapeutic value of cannabis and/or Δ^9^-THC, with or without CBD, in MS patients. In this regard, Sativex’s efficacy has been demonstrated in randomized controlled clinical trials, and its effectiveness has been confirmed in observational studies. It demonstrates relief of symptoms, especially in patients with moderate to severe spasticity who do not respond to initial oral antispastic treatments [50,51,52,53]. However, in 2017, da Rovare and colleagues published a meta-analysis of 16 randomized clinical trials involving 2597 MS patients. They reported that the efficacy of Δ^9^-THC, whether used in combination with CBD or not, in treating spasm frequency and severity is moderate. Additionally, they found low-certainty evidence in spasticity reduction [54]. In another study published this year by Hansen et al., 134 patients (119 with MS (88.9%) and 15 with spinal cord injury (SCI) (11.2%)) were randomized into four groups receiving a placebo (n = 40), THC 2.5 mg (n = 32), CBD 45 mg (n = 31), or THC plus CBD 22.5/45 mg (n = 31) for six weeks, followed by a one-week phaseout. The results indicated that there is no difference between the placebo and treatment with THC or CBD alone or in combination in patients with either MS or SCI experiencing muscle spasticity or neuropathic pain. In addition, no differences were found in secondary and tertiary outcomes, except for more frequent adverse effects in the THC-containing treatment groups [55]. In conclusion, the studies published on this topic are heterogeneous and require careful consideration before drawing definitive conclusions about the effect of THC, CBD, or THC/CBD in MS patients. Some significant variations among the cited studies are in their consideration of factors such as disease severity, coexisting conditions, co-treatments, as well as differences in follow-up duration and formulations [56,57].

## 5. Charcot–Marie–Tooth (CMT)

Charcot-Marie-Tooth (CMT) is one of the most prevalent inherited neuromuscular disorders, affecting approximately 2.6 million people worldwide. It can be caused by mutations in over 100 genes that encode proteins involved in the structure and function of either the peripheral nerve axon or myelin sheath [58]. Typical symptoms of the disease include decreased sensitivity to heat, touch, and pain, as well as weakness, muscle atrophy, reduced ability to move hands, feet, and legs, abnormal gait, and difficulty walking, often leading to frequent stumbling and falling. Deformities of the feet, especially hollow feet, knees, hands, and back, are also common. There are approximately 60 different forms of CMT, classified according to three main criteria: (a) the type of damage to peripheral nerves, determined based on nerve conduction velocities (axonal, demyelinating, or intermediate form); (b) the type of genetic transmission (autosomal dominant, autosomal recessive, or X-linked form); (c) the specific genetic mutation causing the disease. Based on these parameters, CMT is categorized into various types (such as CMT type 1, 2, 4, X-linked, intermediate, etc.) and subtypes (such as CMT1A, 2B, 4C, X1, etc.) [59,60]. Physical and occupational therapy, orthotics, and palliative medications are currently the only options for managing symptoms and slowing the progression of the disease, although new approaches are being developed. This year, Canals et al. published a survey study conducted on 56 patients, 71.4% of whom were female. The average age was 48.9 (SEM = 2.0, Min = 22, Max = 87). Among the subset of participants who reported a known CMT subtype (n = 33), 48.5% had a form of CMT type 1, 27.3% had hereditary neuropathy with liability to pressure palsies (HNPP), 18.2% had CMT type 2, and 6.1% had CMT type 4. In this survey, patients with CMT reported experiencing significant relief from pain symptoms after using medical cannabis, with the majority of them (more than 90%) reporting significant pain relief. Moreover, among the subset of patients taking other medications, 8 out of 10 reduced or interrupted the use of opiates; 11 out of 16 reduced sleep medications; 6 out of 12 reduced anxiolytics; and 11 out of 23 reduced antidepressants [61]. Other preclinical or clinical studies that corroborate these findings are currently not available.

## 6. Peripheral Neuropathy

Chronic peripheral neuropathy (PN) refers to various disorders that affect peripheral nerves. These disorders can be caused by genetic conditions such as amyloidosis, Fabry disease, and CMT disease, as well as by pathological conditions like certain hematological tumors, HIV, diabetes, and diphtheria. Additionally, exposure to toxic substances and anti-cancer drugs can also lead to PN [62]. The onset and course of symptoms vary depending on the PN form. In general, they are classified into three types: motor, sensory, and vegetative. In brief, motor symptoms range from the sensation of awkwardness in the finer movements of fingers to the decreased strength in the large skeletal muscles associated with the sensation of fatigue in carrying out movements. The sensory symptoms are also extremely varied and generally arise slowly, can also be sporadic, and are often initially underestimated, manifesting themselves in the form of more or less intense pain, burning, tingling, and numbness. Neurological signs include an alteration of reflexes, severe or moderate strength deficiency, excessive or absent physical sensitivity (hyper- and hypoesthesia), hyperalgesia (excessive response to mild painful stimuli), or allodynia (painful response to stimuli that normally should not cause pain). Medications to treat PN include steroids, anticonvulsants, immunosuppressants, tricyclic antidepressants, and serotonin–norepinephrine reuptake inhibitors. Despite this armamentarium being further enriched by recently approved biological agents, PN is often refractory to treatments [63,64]. Cannabinoids, endocannabinoids, and synthetic cannabimimetic agents have been extensively documented as powerful agents to control pain sensitization and transmission at both central and peripheral levels for their action on key cellular pathways [65]. In this regard, it was demonstrated that the endocannabinoid system is active in spinal and supraspinal structures regulating the pain sensation with the two major endocannabinoids AEA and 2-AG acting as antinociceptive agents through mechanisms depending on CB1 and CB2 receptors [65,66,67,68,69]. Overall, besides acting on CB1 and CB2 receptors, plant-derived cannabinoids and endocannabinoids are known to regulate the pain sensation and response through many other targets including G protein-coupled receptor (GPCR) 55, GPR18, opioid and serotonin receptors, PPARs, cys loop ligand-gated ion channels, and transient receptor potential (TRP) channels (TRPV1, TRPA, and TRPM subfamilies) [70,71,72]. Hence, the complex polyvalent pharmacological proprieties of cannabinoids, endocannabinoids, and derivates in pain mechanisms have been explored in a large number of preclinical studies [66,70,73,74].

Large amounts of evidence tend to support the use of smoked or inhaled Cannabis as a monotherapy or add-on therapy against PN in humans [70,75]. In this regard, in 2008, Wilsey et al. conducted a double-blind, placebo-controlled crossover study on 38 patients, of which 22 were diagnosed with complex regional pain syndrome, 10 had central neuropathic pain, and 6 had peripheral neuropathic pain, to evaluate the analgesic efficacy of cannabis. In this study, participants were assigned to smoke high-dose cannabis cigarettes (7%), low-dose (3.5%) cannabis cigarettes, or a placebo and invited to complete a cumulative dosing procedure. The investigators found that 3.5% Δ^9^-THC and 7% Δ^9^-THC were equally efficacious in reducing the intensity of pain with minimal and well-tolerated psychoactive and cognitive effects observed only at higher doses [76]. In this line, Ellis et al. found that smoked cannabis (containing 1–8% Δ^9^-THC) significantly reduced pain intensity in patients with HIV-associated distal sensory predominant polyneuropathy (DSPN) who were unresponsive to analgesics [77]. Ware et al. performed a small trial including 21 patients affected by post-traumatic or post-surgical neuropathic pain and randomly assigned to receive cannabis in four different formulations (containing 0%, 2.5%, 6.0%, and 9.4% Δ^9^-THC) for 14 days. Daily average pain intensity was measured using a numeric rating scale. The investigators also assessed secondary outcomes such as mood, sleep, quality of life, and adverse effects. Patients with the highest content of Δ^9^-THC (9.4%) were the only ones to report a significant reduction in pain sensation and sleep latency, despite the investigators finding that it was only modest (pain reduction 0.7 from 6.1 to 5.4 on a 10 cm scale). Adverse effects included throat irritation, burning sensation, headache, dizziness, and fatigue, and some cases were aggravated with higher doses of Δ^9^-THC [78]. Wilsey et al., in 2013, in another double-blind, placebo-controlled crossover study, analyzed the effect of vaporized cannabis in 39 patients with central and peripheral neuropathic pain resistant to conventional pharmacotherapies who underwent a standardized procedure for inhaling medium-dose (3.53%), low-dose (1.29%), or placebo cannabis with the primary outcome being visual analog scale pain intensity. Both the low and medium doses provided a statistically significant reduction (30%) in pain intensity when compared to that provided by the placebo. Undesirable consequences were similar to those reported in the studies mentioned above (i.e., psychological and/or cognitive effects) but also in this case were generally tolerated by patients [76]. In numerous other clinical studies, cannabis, cannabinoids, or synthetic analogs were proven to exert analgesic effects against chronic peripheral neuropathies [79,80,81,82]. Notwithstanding, in 2018, Stockings et al. reported data from a systematic meta-analysis performed on 91 publications containing 104 studies (n = 9958 participants), including 47 randomized controlled trials (RCTs) and 57 observational studies concluding that the effectiveness of cannabinoids in chronic forms of chronic forms of non-cancer pain is only modest [83]. This year, Solmi et al. published another umbrella review of 101 meta-analyses of randomized controlled trials and observational studies discussing the risks and benefits of cannabinoids in different forms of chronic pain. They found that there is convincing and convergent evidence that cannabis and cannabinoids (THC in association or not with CBD) could also be considered effective against chronic pain across different conditions; however, caution is necessary since key aspects were underestimated, for instance, the type of cannabis smoked, cost-effectiveness, and most importantly, harmful effects of long-term therapies that remain poorly known [84,85].

## 7. Myasthenia Gravis

Myasthenia gravis (MG) is a chronic autoimmune disease affecting the neuromuscular junction (NMJ). It is caused by the abnormal presence of circulating antibodies directed against the acetylcholine (AChR) and tyrosine kinase (MuSK) muscle receptors. Low-density lipoprotein receptor-related protein 4 is an additional target of autoantibodies. The thymus is believed to be responsible for producing anti-AChR antibodies, which have been demonstrated to play a pathogenetic role in all forms of the disease. MG can also be caused by drugs such as D-penicillamine and interferon-alpha, as well as Epstein–Barr virus infection. Autoantibodies impair the physiological neurotransmission of contractile signals sent from spinal nerves to muscles, resulting in fluctuating levels of weakness and fatigue that quickly appear and worsen when certain muscle groups are in use [86]. Therapeutic options are extremely limited. Medications like cholinesterase inhibitors, corticosteroids, and immunosuppressants may only help alleviate symptoms [87].

Cannabinoids and endocannabinoids play an important role in regulating neuromuscular transmission. In this regard, Van der Kloot et al., using frog neuromuscular junction preparations, found that AEA (10 µM) did not affect the basal miniature endplate potential (mEPP) frequency. However, interestingly, when frequency was increased in the presence of a cAMP agonist, AEA was able to restore mEPPs to resting levels [88]. Technically, the mEPP frequency is assumed to be an indirect measure of presynaptic ACh release at the NMJ. In another study, Tarasova et al., via the preparation of a mouse diaphragm and m. extensor digitorum longus, demonstrated that AEA (30 µM) increased mEPP frequency and quantal content (QC), but not amplitude, in a manner prevented by the L-type Ca^2+^-channel blocker nitrendipine (1 µM). In addition, 2-AG (1 µM) was also found to increase mEPP amplitude and QC, but not their frequency. Remarkably, these 2-AG effects were abolished only in the presence of PKA inhibitors, revealing that the mechanism by which the two endocannabinoids control mEPP is different [89]. In a previous study, Newman et al. discovered that 2-AG mediates the muscarine M3 receptor-dependent inhibition of ACh release from nerve terminals through the same “retrograde mechanism” that occurs in the CNS. Furthermore, the same researchers found that the stimulation of CB1 significantly reduced the peak of transient calcium in motor neuron terminals, which is necessary to activate the intracellular cascades for the release of ACh [90]. In the same vein, Sanchez-Pastor et al. demonstrated that CB1 receptors, and not CB2 receptors, are responsible for the inhibition of mEPP frequency at the frog’s end plate [91]. A few years later, Silveira et al. published an interesting study demonstrating that WIN 55212-2 and ACEA had opposing effects on evoked quantal ACh release, revealing the role of TRPV1 receptors in the effects produced by synthetic cannabinoids at the NMJ [92]. In other studies, the results obtained with Δ^9^-THC are conflicting [93,94,95].

In a recent study, Puopolo et al. found that certain plant cannabinoids, such as CBD, Δ^8^-THC, CBG, CBGA, CBT, CBDV, CBC, and CBN, exhibited moderate inhibitory effects on the activities of acetylcholinesterase (AChE) and butyrylcholinesterase (BChE) enzymes. These effects may contribute to their modulatory influence on the cholinergic system in the neuromuscular junction (NMJ) [96]. Cannabinoids and endocannabinoids, in a manner that depends on and is independent of CB1 receptors, are also known to play a role in suppressing the release of calcium from the sarcoplasmic reticulum, thereby inhibiting excitation–contraction coupling [95,97,98]. In 2018, Morsch et al., using a mouse model of myasthenia gravis generated via daily injections of IgG from an anti-MuSK-positive MG patient, demonstrated for the first time that acute treatment with WIN 55212 is beneficial for rescuing disease-impaired neuromuscular transmission [98]. Apart from these findings, no other studies have been published on this topic.

## 8. Muscular Dystrophies

Muscular dystrophies are a group of over 30 inherited disorders caused by mutations in genes that encode proteins responsible for structural functions in skeletal muscles. As a result, the malfunctioning of these proteins is responsible for the progressive weakness and degeneration of muscle tissues. Among them, Duchenne and Becker muscular dystrophies (DMD—BMD) are the most common forms, with a prevalence ranging from 19.8 to 25.1 per 100,000 person years [99]. Both diseases are caused by mutations in the same gene located in the short arm of the X chromosome (Xp21.2 locus), which provides instructions for making dystrophin, a 427-kDa cytoskeletal protein that, in complex with other associated proteins belonging to the dystrophin-associated protein complex (DAC), serves to physically connect the cytoskeleton of a muscle fiber to the surrounding extracellular matrix through the cell membrane [100]. Due to the X-linked inheritance pattern, boys are predominantly affected. However, despite being caused by alterations in the same gene, DMD is associated with more severe progression and prognosis than is BMD. The reason is that in DMD, unlike BMD, dystrophin functionality is more compromised. The primary pathological feature of DMD is the progressive and unstoppable degeneration of skeletal muscles, which initiates in the patients’ early years of life. Other severe complications include difficulties in breathing and swallowing, lumbar lordosis, cardiomyopathy, and varying degrees of intellectual disability [101,102]. For DMD, as with other types of muscular dystrophy, there is currently no cure, although numerous experimental approaches are being investigated [103]. To date, very little is known about the potential use of cannabis and cannabinoids in the treatment of skeletal muscle dystrophies. In recent years, our research group has conducted pioneering studies showing that the endocannabinoid system is dysregulated in skeletal muscles of mdx (muscular dystrophy X-linked) mice, a validated preclinical model of DMD, as well as in muscles of DMD donors. In particular, we found that the expression of the CB1 gene changes during the progression of the disease, reaching peak levels at the onset. Additionally, using bioinformatics and biochemical tools, we discovered that the CB1 gene is controlled by PAX7, a key muscle-specific transcription factor that regulates the self-renewal, proliferation, and commitment of satellite cells [104,105]. This study followed a previous one in which we reported changes in endocannabinoid system (ECS) activity during physiological muscle formation in both in vivo and in vitro models. In the same study, we found that the selective antagonism of CB1 receptors by Rimonabant (or SR141716) or AM251 inhibited the proliferation of both murine and human myoblasts, and conversely promoted their differentiation into mature myotubes. In contrast, an opposite effect was observed with ACEA and Noladin ether, which are synthetic selective agonists of CB1 [106]. Taken together, these findings led us to hypothesize that the increased expression of CB1 in muscle dystrophic mice and humans could contribute to the reduced ability of muscle precursor cells to terminate their differentiation, hence negatively affecting the regeneration process [107]. In support of this hypothesis, mdx mice treated with Rimonabant showed (i) a higher number of regenerated myofibers; (ii) reduced expression of systemic and tissue-specific markers of inflammation; (iii) restoration of muscle strength and locomotor activity. In contrast, in dystrophic mice treated with ACEA, we observed that all these parameters were worsened [105]. It is worth emphasizing that Rimonabant was the first cannabinoid-based drug to be marketed for the treatment of obesity in 2006. However, it was withdrawn from the market a few years later due to serious psychiatric side effects [108]. Remarkably, in our study, we proposed a “peripheral” use of Rimonabant, providing an opportunity for its repurposing in the market and potentially bypassing the lengthy time required for new drug development [105]. In a study published this year, we once again demonstrated that mdx mice have elevated circulating levels of endocannabinoids, which are associated with gut dysbiosis and a reduced abundance of beneficial bacterial species, including those that produce short-chain fatty acids, primarily acetate, propionate, and butyrate. Notably, supplementing mdx mice with sodium butyrate resulted in reversing deficits in locomotor activity, reactivating muscle autophagy, and preventing inflammation associated with excessive endocannabinoid signaling at CB1 receptors in muscle cells. This effect depended on the activation of GPR109A and PPARg receptors. Strikingly similar results were obtained in primary muscle cells from donors with DMD [109]. Major phytocannabinoids, including CBD, CBDV, and THCV, also produced significant effects in the treatment of DMD. In this context, in 2019, we reported that CBD and CBDV promoted the differentiation of murine C2C12 myoblast cells into myotubes by primarily increasing [Ca^2+^]_i_ through TRPV1 activation. Notably, in primary satellite cells and myoblasts isolated from healthy and/or DMD donors, not only CBD and CBDV but also THCV promoted myotube formation, primarily through TRPA1 activation. In mdx mice treated with CBD or CBDV (60 mg kg^−1^), locomotor activity was restored, accompanied by reduced muscle inflammation and restored autophagy [110]. Recently, Argenziano et al. found a decreased expression of CB2 receptors in DMD-associated macrophages. They also observed a beneficial effect of the selective agonist JWH-133 in reducing inflammation. This was achieved by inhibiting the release of pro-inflammatory cytokines and by shifting macrophages from a proinflammatory M1 to an anti-inflammatory M2 phenotype [111]. However, despite the encouraging results discussed above, there are currently no ongoing clinical trials. Further preclinical research conducted in both in vitro and animal models is essential for predicting the effectiveness of cannabinoid therapies, not only in DMD but also in other rare dystrophies and non-dystrophic myopathies.

## 9. Conclusions

Preclinical studies have shown that cannabis with varying concentrations of Δ^9^-THC, as well as the single administration of Δ^9^-THC with or without CBD, is effective to varying degrees in relieving symptoms of common neuromuscular diseases. This is due to the wide range of anti-oxidant, anti-inflammatory, and neuroprotective properties. However, the evidence obtained from clinical trials sometimes dampens enthusiasm. For example, cannabis-based medicines have generated significant interest among multiple sclerosis (MS) patients for alleviating pain and muscle spasticity. However, recent meta-analyses have shown that the beneficial effects of cannabinoids in addressing these symptoms are only modest, often being comparable to those observed in patients treated with a placebo. Furthermore, there is encouraging preclinical evidence suggesting that cannabis and cannabinoids are potent tools for treating various types of central and peripheral pain. However, clinical studies have not only failed to demonstrate that cannabis or Δ^9^-THC relieves pain, but have paradoxically reported an increased sensitivity to pain in voluntary patients [112]. Low doses of THC, which are typically used in the majority of clinical studies, appear to have an overall safe profile concerning psychiatric and other severe symptoms. In contrast, in studies conducted with higher doses of THC (>10%), significant risks to the executive function and motor control of patients were observed [113,114].

However, there are still significant limitations in clinical studies, including the absence of information on the pharmacokinetics, pharmacodynamics, safety, and tolerability of long-term administration of cannabis-based medicines. Another critical issue is the small sample size, as well as the frequent inadequate comparison between cannabis-based medicines and conventional drugs. For many other neuromuscular disorders (NMDs), clinical trials have not been conducted at all, although the data obtained in preclinical models are encouraging. This is, for example, the case of DMD. In some cases, the potential use of cannabinoids has not been explored, not even at a preclinical level. In conclusion, the potential benefits of cannabis-based medicines should not be overestimated or underestimated. Preclinical and clinical studies must meet all efficacy requirements. Long-term risks must be carefully considered, particularly in the most vulnerable patients, such as adolescents or those with psychiatric or cardiac disorders. Great attention must be paid to the compassionate use of cannabis, which remains controversial, generates concerning scientific data, and raises numerous safety and public health issues [115]. It is also advisable to be cautious when using cannabis in combination with other drugs [116].

## Data Availability

Not applicable.
